# Frameless robotic stereotactic brain biopsy workflow with CT-MRI fusion and CT-to-fluoroscopy registration: Step-by-step technical note and early experience

**DOI:** 10.1016/j.bas.2026.105940

**Published:** 2026-01-12

**Authors:** Mario Taravilla-Loma, Víctor Rodríguez-Domínguez, Catalina Vivancos Sánchez, María Luisa Gandía-González, Alberto Isla Guerrero

**Affiliations:** Department of Neurosurgery, Hospital Universitario La Paz, Paseo de la Castellana 261, 28046, Madrid, Spain

**Keywords:** CT-to-fluoroscopy registration, CT-MRI fusion, Intraoperative fluoroscopy, Frameless stereotaxy, Robot-assisted brain biopsy, ExcelsiusGPS

## Abstract

**Introduction:**

Stereotactic brain biopsy is a widely used procedure for the histopathological and molecular diagnosis of different types of brain lesions. While frame-based techniques remain highly accurate, frameless neuronavigation and robotic platforms have progressively streamlined workflow and standardization. Practical, reproducible registration strategies are key to facilitate safe implementation across centers.

**Research question:**

We describe a frameless, robot-assisted stereotactic brain biopsy workflow based on preoperative CT-MRI fusion and intraoperative fiducial-free CT-to-fluoroscopy registration, and report technical considerations for reproducible adoption.

**Material and methods:**

A robot-assisted brain biopsy was performed on a patient with a right frontal butterfly-shaped lesion, based on CT-MRI fusion and CT-to-fluoroscopy “Merge Images” registration. We detail the step-by-step workflow, including the registration strategy, technical advantages and disadvantages, and our initial experience with this protocol.

**Results:**

CT-to-fluoroscopy registration provided reliable intraoperative anatomical correlation and was integrated into the routine setup without intraoperative CT or cone-beam CT. In the index case (and in our early experience), tissue sampling provided a conclusive histomolecular diagnosis, with procedure times consistent with routine stereotactic biopsy practice and no major procedure-related complications.

**Discussion and conclusion:**

This technical note outlines a reproducible, step-by-step workflow for robot-assisted stereotactic brain biopsy based on CT-MRI fusion and CT-to-fluoroscopy registration, supported by a standardized operating-room setup. We highlight the practical checkpoints that keep the procedure reliable in routine use, particularly strict fusion verification and uninterrupted optical tracking. Further experience and larger series are warranted to refine its role alongside established stereotactic techniques.

## Highlights

• Micro-burr hole robot-assisted brain biopsy with ExcelsiusGPS.
• CT-MRI fusion and CT-to-fluoroscopy registration enable a simple, fiducial-free, streamlined workflow.
• Step-by-step workflow using the ExcelsiusGPS “Merge Images” CT-to-fluoroscopy registration.
• Early experience supports feasibility: conclusive diagnosis, no major complications, and ∼24-h stay.
• Trajectory locking may reduce operator variability compared with manual frameless biopsy.

## 1 Introduction

Stereotactic brain biopsy is an established method to obtain tissue diagnosis of deep or diffuse brain lesions (Bex and Mathon, 2023; Li et al., 2025). For decades, rigid frame brain biopsy has been considered the gold standard in terms of accuracy, with a diagnostic yield of around 95 %, but it can add patient burden and workflow complexity (Gecici et al., 2025; Hu et al., 2022). Subsequently, with the advent of frameless neuronavigation, diagnostic yield became comparable to frame-based approaches while operative time decreased (Bex and Mathon, 2023).

Robot-assisted brain biopsy further supports workflow standardization and reproducibility, with published series reporting high diagnostic yield, submillimetric targeting accuracy, and safety profiles comparable to frame-based and frameless approaches (Gecici et al., 2025; Hu et al., 2022; Porto Junior et al., 2024; Chuck et al., 2024; Feng et al., 2023). At the same time, it is important to acknowledge that many “minimal-access” elements—such as puncture-type scalp openings, very small burr holes and single-stitch closure—have been described across frame-based, frameless neuronavigation-guided, and robot-assisted techniques (Mathon et al., 2025; Mallereau et al., 2025). However, in routine frameless practice, the most commonly used approach remains a short linear incision and a standard trephine hole (Li et al., 2025; Gecici et al., 2025; Hu et al., 2022; Chuck et al., 2024; Feng et al., 2023). In this context, protocols that reliably combine minimal cutaneous disruption with a highly reproducible registration strategy and operating-room (OR) setup are particularly attractive when the goal is to simplify the procedure without compromising stereotactic discipline or diagnostic performance.

In this technical note, we present a detailed step-by-step workflow for frameless, robot-assisted stereotactic brain biopsy using the ExcelsiusGPS cranial platform, based on preoperative CT-MRI fusion and intraoperative CT-to-fluoroscopy “Merge Images” registration. We detail the technique through an index case and provide a pragmatic protocol aimed at facilitating reproducibility, together with practical setup pearls and pitfalls that focus on registration quality, fluoroscopic views, and optical tracking conditions. Finally, we contextualize this workflow within the current landscape of framed, frameless and robotic biopsies, including brief considerations about the learning curve based on recent evidence and our early experience.

## 2 Material and methods

### 2.1 Case description

**Fig. 1:**
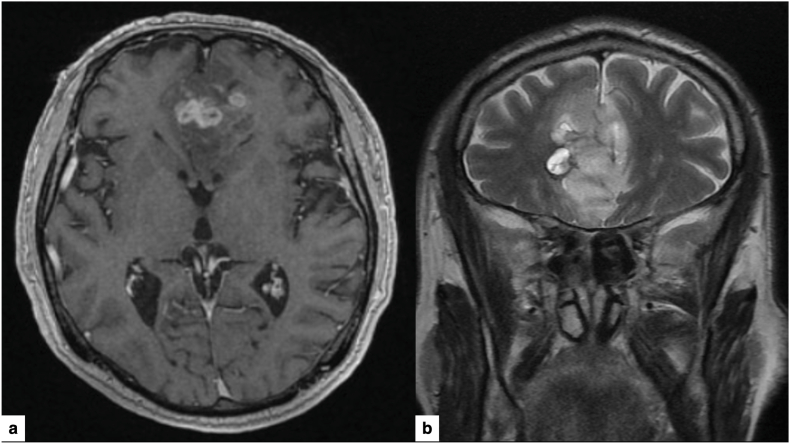
Preoperative MRI. (a) Axial T1-weighted post-contrast image showing a bilateral frontal “butterfly” lesion crossing the corpus callosum. (b) Coronal T2-weighted image demonstrating the infiltrative nature and extent of the lesion.

A 64-year-old right-handed man came to the Emergency Department with symptoms of memory impairment, apathy, and intermittent headaches that had been going on for three months. Magnetic resonance imaging (MRI) revealed a poorly defined lesion affecting both frontal lobes and crossing the corpus callosum (butterfly pattern), with slight peripheral enhancement on post-contrast T1 and hyperintensity on T2/FLAIR, which was radiologically consistent with a high-grade glioma (Fig. 1). Given the deep, bilateral, and diffuse involvement, a multidisciplinary tumor committee decided to perform a robot-assisted brain biopsy to obtain a definitive diagnosis of the lesion. The case was considered suitable for this workflow because rigid head fixation, true orthogonal AP/lateral fluoroscopy, and uninterrupted optical tracking could be reliably achieved with the planned operating-room setup (Chuck et al., 2024).

### 2.2 Surgical planning

**Fig. 2:**
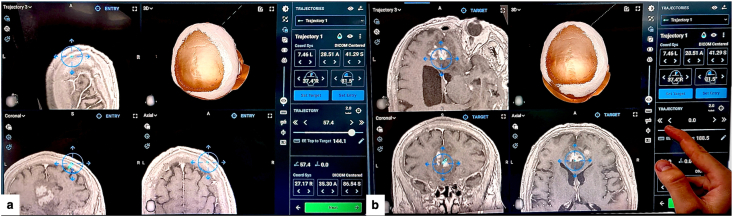
Robotic planning of the stereotactic trajectory (ExcelsiusGPS). (a) CT-MRI fusion and 3D reconstruction showing a right frontal entry point providing the shortest safe, near-perpendicular corridor avoiding eloquent cortex and superficial vessels. (b) Target positioned within the most representative component of the lesion, defining a coaxial trajectory through a micro-burr hole of approximately 57.4 mm.

The trajectory was planned using the ExcelsiusGPS platform, importing high-resolution volumetric CT and MRI brain scans (thin slices, ≤1 mm) performed preoperatively. After planning an appropriate operating room layout that would allow truly orthogonal AP/lateral fluoroscopy and uninterrupted optical tracking throughout the workflow, right frontal entry point was selected to avoid the eloquent cortex and superficial/penetrating vessels along the path, while calculating the shortest possible distance to the lesion (Hu et al., 2022; Chuck et al., 2024). Whenever possible, the biopsy trajectory was planned to be as perpendicular as possible to the calvarial surface to minimize skiving and reduce target point error (TPE). Subsequently, the target point was planned in the T1-enhancing component of the right frontal part of the lesion to maximize diagnostic yield (Li et al., 2023). The length of the planned intracranial trajectory was ∼57.4 mm from the entry into the skull to the target (Fig. 2).

### 2.3 Surgical technique and workflow

#### 2.3.1 Workflow prerequisites and less suitable scenarios

This workflow is best suited when the patient can undergo rigid head fixation and the team can obtain true orthogonal AP/lateral fluoroscopy with a stable line-of-sight for optical tracking. It may be less advisable when orthogonal fluoroscopy is unreliable, when optical tracking is frequently interrupted, or when patient positioning would force a non-ergonomic trajectory or limited access to the entry site; in such cases, alternative frameless or frame-based strategies may be preferred (Chuck et al., 2024).

#### 2.3.2 Patient positioning and setup

**Fig. 3:**
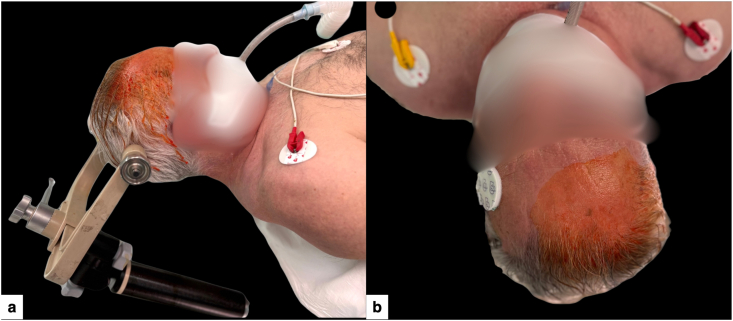
Patient positioning for minimal-access robot-assisted stereotactic biopsy. (a) Supine positioning with the head fixed in a three-pin Mayfield skull clamp and slightly rotated to elevate the right frontal entry region, facilitating a near-perpendicular trajectory for the planned micro-burr hole and unobstructed optical tracking. (b) Close-up of the prepared entry area, with a small exposed field centered over the planned puncture site.

Under general anesthesia, the patient was placed in the supine position, with the head slightly rotated to elevate the right frontal entry region and secured in a three-pin Mayfield skull clamp (Fig. 3), allowing a near-perpendicular calvarial trajectory, true orthogonal AP/lateral fluoroscopy, and a stable optical line-of-sight to the DRB, end-effector, and instruments throughout the procedure (Hu et al., 2022; Chuck et al., 2024).

#### 2.3.3 Intraoperative fluoroscopy and CT-to-fluoroscopy image fusion

**Fig. 4:**
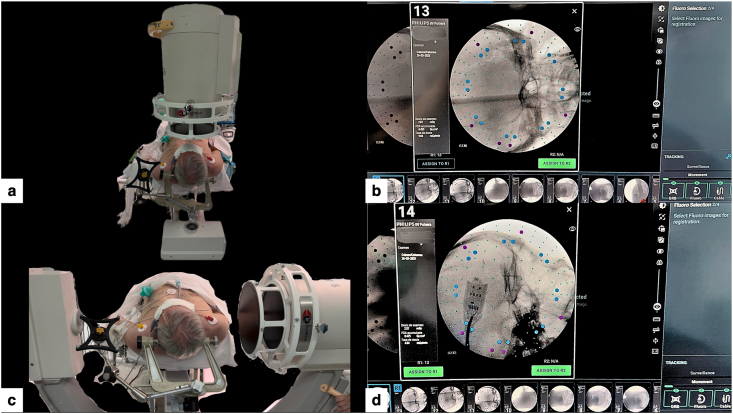
CT-to-fluoroscopy acquisition for ExcelsiusGPS registration. (a) Intraoperative setup with the C-arm positioned for orthogonal anteroposterior cranial fluoroscopy, with the dynamic reference base (DRB) in view. (b) Anteroposterior fluoroscopic image selected for registration, showing clear visualization of skull base bony landmarks. (c) C-arm positioned for true lateral cranial fluoroscopy while maintaining an unobstructed line-of-sight to the DRB. d) Lateral fluoroscopic image selected for registration, providing complementary landmarks for subsequent CT-to-fluoro “Merge Images” alignment.

The registration was performed using a CT-to-fluoroscopy “Merge Images” workflow. Two orthogonal cranial fluoroscopic images (anteroposterior and lateral skull views) were acquired and fused with the preoperative cranial CT dataset (Fig. 4), to which the brain MRI had been fused for trajectory planning (Hu et al., 2022; Chuck et al., 2024). This workflow enabled fiducial-free registration that, in our routine setup, was typically completed in <20 min, provided that high-quality orthogonal views were obtained and fusion was strictly verified; acquisition and fusion were repeated whenever anatomical concordance was not judged excellent (Hu et al., 2022; Chuck et al., 2024).

**Fig. 5:**
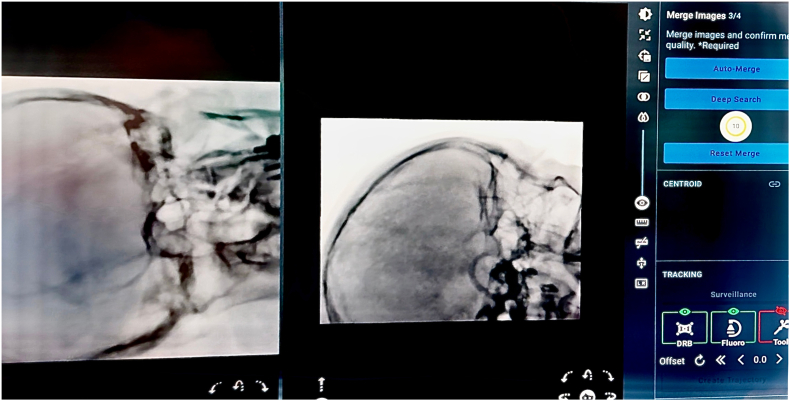
“Merge Images” fusion verification (ExcelsiusGPS). CT-to-fluoroscopy “Merge Images” verification screen on the ExcelsiusGPS platform, showing alignment of skull base bony landmarks and a merge quality score of 10/10, confirming accurate image fusion.

After image registration and fusion (Fig. 5), the team verified the anatomical concordance between the navigated probe and the bony landmarks (Chuck et al., 2024; Li et al., 2023). Once the anatomical verification was deemed satisfactory and the entry point was identified on the scalp, in preparation for the subsequent step of skin disinfection.

#### 2.3.4 Micro-burr hole and biopsy sampling

**Fig. 6:**
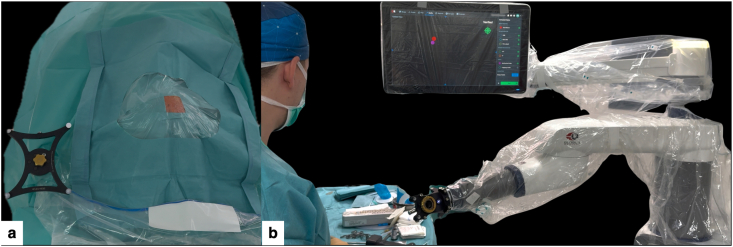
Sterile setup and robotic docking for minimal-access biopsy. (a) Standard sterile surgical field exposing only the planned entry site and the sterile dynamic reference base (DRB), limiting the cranial exposure while preserving optimal tracking. (b) ExcelsiusGPS robotic arm fully draped with the sterile end-effector mounted and navigation instruments verified, ready to automatically align with the planned trajectory.

A standard sterile surgical field was prepared, exposing only the planned entry point and the sterile dynamic reference base (DRB) (Fig. 6a).

The sterile robotic end-effector (instrument holder, [EE]) was then mounted on the ExcelsiusGPS arm and verified with the navigation system (Fig. 6b).

Once registration and instrument verification were completed, the robotic arm automatically moved to the pre-planned trajectory and was locked in position over the marked entry point (Supplementary Video 1).

Supplementary data related to this article can be found online at https://doi.org/10.1016/j.bas.2026.105940

The following are the Supplementary data related to this article.

Multimedia component 1 ***Supplementary Video 1***. ExcelsiusGPS robotic arm automatically moving to the pre-planned right frontal trajectory. The system reaches optimal alignment (green indicator on the navigation screen), confirming coaxial positioning over the planned entry site. Multimedia component 1

**Fig. 7:**
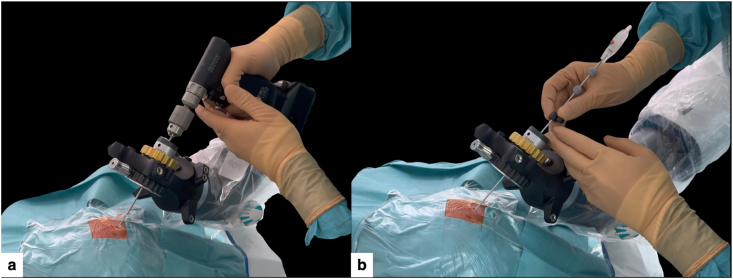
Micro-burr hole creation through a punctiform scalp opening and navigation-guided biopsy. (a) A 2.7 mm drill advanced through the sterile end-effector under ExcelsiusGPS guidance, perpendicular to the calvarial surface, creating a micro-burr hole through a punctiform scalp opening, avoiding a conventional linear incision. (b) A 2.1 mm stereotactic biopsy needle introduced through the same aligned end-effector and micro-burr hole corridor, advancing under continuous real-time navigation towards the planned target.

Through a punctiform scalp opening (without a linear incision), a 2.7 mm helical drill was advanced under robotic guidance, perpendicular to the cranial surface and collinear with the planned trajectory (Fig. 7a), to reduce skiving and target point error (Hu et al., 2022; Chuck et al., 2024; Li et al., 2023).

After establishing the bone and dural corridor, a 2.1 mm outer-diameter stereotactic biopsy needle was inserted through the robotic end effector (Fig. 7b).

Active navigation provided continuous real-time feedback of the needle position relative to the planned path, while the robotic arm maintained rigid coaxial alignment to avoid deviation (Supplementary Video 2), advancing the needle to the predetermined target depth (∼57 mm from skull entry). Multiple cores were obtained from the enhancing portion of the lesion using the standard twist-and-cut technique (Bex and Mathon, 2023; Porto Junior et al., 2024; Feng et al., 2023). The needle was then withdrawn along the same path under navigation control.

Supplementary data related to this article can be found online at https://doi.org/10.1016/j.bas.2026.105940

The following are the Supplementary data related to this article.

Multimedia component 1 ***Supplementary Video 2***. Insertion of the stereotactic biopsy needle through the sterile end-effector along the planned trajectory, with continuous real-time navigation feedback on the ExcelsiusGPS screen. Robotic trajectory locking supports controlled advancement to the target. Multimedia component 1

**Fig. 8:**
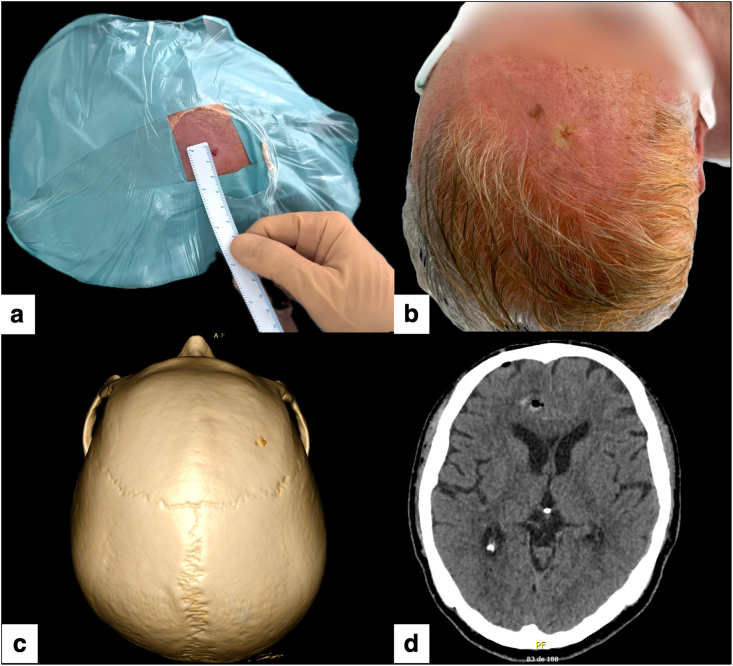
Postoperative wound appearance, micro-burr hole visualization, and radiological control. (a) Immediate postoperative view showing a single pinpoint entry site of only a few millimeters, without a conventional scalp incision. (b) Final aspect of the minimal-access robot-assisted biopsy, showing a single fine suture and no visible linear incision. (c) 3D CT reconstruction revealing the 2.7 mm micro-burr hole on the right frontal bone, barely visible on the skull surface. (d) Axial postoperative CT scan confirming correct biopsy trajectory and absence of significant hemorrhage or mass effect.

Due to the narrow working channel, hemostasis was achieved after brief observation at the entry site, without the need for additional hemostatic agents. A single fine resorbable suture was placed to approximate the tiny puncture. Early postoperative 3D reconstruction and axial CT confirmed the right frontal micro-burr hole, correct biopsy trajectory and absence of significant hemorrhage or mass effect (Fig. 8a–d).

### 2.4 Step-by-step workflow summary (Table 1)

**Table 1:** Step-by-step workflow for frameless ExcelsiusGPS-guided stereotactic brain biopsy using CT-to-fluoroscopy “Merge Images” registration.

Step	Description	Key technical points
*1. Preoperative imaging and planning*	Obtain a high-resolution MRI with contrast and a thin-slice (≤1 mm) CT scan. Plan the entry point and target point.	• Select a short and safe trajectory, avoiding the eloquent cortex, sulci, and major vessels. • Attempt an entry that is nearly perpendicular to the skull. • Set the target in the most representative area of the lesion (contrast-enhancing or metabolically active region).
*2. Case selection and OR feasibility check*	Confirm that the planned trajectory can be executed with a comfortable head position and an operating-room layout that enables true AP/lateral fluoroscopy and uninterrupted optical tracking (robot arm, C-arm, camera, DRB).	• Case selection = setup feasibility: Prefer cases in which the head can be fixed neutral or with mild rotation while still allowing truly orthogonal AP and lateral fluoroscopy. • Prior OR geometry check: Confirm C-arm sweep, robotic arm reach, and uninterrupted optical line-of-sight to the DRB/end-effector/instruments.
*3. Patient positioning and head fixation*	Position the patient so that the robotic arm can move comfortably and stably (supine, lateral, or prone position, as needed) and secure the head with a three-pin Mayfield skull clamp	• Ensure ergonomic access for the surgeon and robot. • Place a radiolucent/appropriately positioned Mayfield clamp that does not obstruct true AP/lateral fluoroscopy.
*4. Image registration and fusion with preoperative imaging*	Acquire orthogonal AP and lateral fluoroscopic views and merge them with the preoperative CT–MRI dataset in the robotic navigation software (“Merge Images”).	• Acquire high-quality orthogonal AP and lateral views and merge them with the preoperative CT–MRI dataset (“Merge Images”). • Accept only excellent fusion; repeat fluoroscopy acquisition and fusion if any mismatch is detected.
*5. Anatomy check and entry marking*	Perform an anatomy check with a navigated probe to verify concordance between virtual images and real bony landmarks; once confirmed, mark the final entry point on the scalp.	• Proceed only with perfect concordance: adjust and re-verify if mismatch is detected. • Mark the single entry point (the robot should subsequently align itself toward that point).
*6. Sterile setup, instrument selection and calibration*	Prepare the sterile field, drape the robot, mount the sterile end-effector, and calibrate the drill and biopsy needle.	• Use a small drill bit (<3 mm) compatible with the ∼2.1 mm biopsy needle. • Set a depth stop: drill just beyond the inner table (≈1–2 mm) to open dura while avoiding overpenetration.
*7. Robotic alignment to planned trajectory*	Command the robot to move automatically to the planned trajectory and lock over the marked entry.	• Confirm correct alignment on the ExcelsiusGPS screen (trajectory status in green). • Verify uninterrupted optical tracking of the DRB, end-effector, and instruments. • Verify that the tool axis is colinear with the planned path and that no mechanical conflicts exist.
*8. Minimal-access small-diameter hole creation*	Create a pinpoint skin puncture and advance a small-diameter drill (≈2.5–3 mm) through the end-effector to form a small-diameter hole along the planned axis.	• Use a punctiform scalp opening. • Keep the drill aligned with the planned axis to minimize skiving. • Drill just enough to cross bone and dura without overpenetration.
*9. Navigation-guided biopsy sampling*	Introduce a stereotactic biopsy needle (≈2.1 mm) through the end-effector and advance under continuous navigation to the target.	• Use real-time tracking and robotic stability to prevent deviation. • Obtain multiple cores from the lesion region to optimize diagnostic yield.
*10. Needle withdrawal and closure*	Withdraw the needle along the same tract under navigation; inspect the entry site. Close the puncture with a single fine suture.	• The narrow tract usually requires only gentle compression, reserve hemostatic agents for exceptional cases. • Recommended suture to close skin tightly (dura mater is open).

## 3 Results and discussion

In the illustrative case, robot-assisted stereotactic biopsy achieved a definitive diagnosis of IDH-wildtype glioblastoma on the first pass, providing sufficient tissue for a complete histomolecular profile. The procedure was completed through a single micro-burr hole*,* with no neurological deficits and no clinically relevant bleeding on postoperative imaging, allowing discharge within 24 h.

Our early institutional experience using the same standardized ExcelsiusGPS workflow has been consistent with the index case. In this small initial series (n = 10), tissue sampling provided a conclusive diagnosis in all cases, with procedure times of approximately 50–80 min and hospital stays close to 24 h. No major complications were observed; minor events such as small, asymptomatic hemorrhage on imaging were managed conservatively. Fluoroscopy-based registration was completed in less than 20 min, without the need for intraoperative CT or cone-beam CT. No direct comparison has been performed and these observations should be interpreted descriptively.

Although the number of patients is limited and no formal statistical analysis is presented, the reproducibility across these early cases supports the feasibility of this workflow in routine clinical practice.

### 3.1 Diagnostic performance and safety

Studies based on robot-assisted brain biopsies using systems such as Remebot, Neuromate, and others report diagnostic yields in the range of 94–100 % and low associated morbidity and mortality rates, comparable to frame-based and frameless techniques (Bex and Mathon, 2023; Gecici et al., 2025; Hu et al., 2022; Porto Junior et al., 2024; Feng et al., 2023; Li et al., 2023).

Recent studies have evaluated brain biopsies performed with the ExcelsiusGPS robot (Chuck et al., 2024), demonstrating submillimeter accuracy (approximately 0.6–0.7 mm), comparable to other high-precision stereotactic systems.

Our experience supports this evidence: diagnostic success without a higher rate of associated complications, despite using a minimal-access approach. These findings suggest that, with rigorous planning and navigation, reducing the size of the access corridor does not appear to compromise diagnostic performance or safety. In addition, by constraining the instrument axis to a planned trajectory, robotic guidance may help reduce operator-dependent variability during needle advancement.

### 3.2 Workflow, registration strategy and patient-centered benefits

Our protocol involves a key element: preoperative CT–MRI fusion with intraoperative CT-to-fluoroscopy “Merge Images” registration. This workflow enables rapid, fiducial-free registration while maintaining reliable anatomical correlation, without requiring intraoperative CT (iCT) or cone-beam CT (CBCT).

Published series using different robotic registration strategies suggest that fluoro-based and iCT/CBCT-based workflows can achieve high accuracy, and are at least as reliable as classic fiducial-based systems (Hu et al., 2022; Chuck et al., 2024; Li et al., 2023). In this context, the CT-MRI plus CT-fluoroscopy workflow we describe offers a lighter and easier-to-adopt option, although direct comparative trials are not yet available. This workflow is best suited to cases in which true orthogonal AP/lateral fluoroscopy and uninterrupted optical tracking can be ensured. Compared with conventional frameless neuronavigation, where the trajectory is often manually maintained, robotic trajectory locking may reduce operator dependence and procedural variability, provided that registration quality and optical tracking conditions are strictly ensured.

Furthermore, for the patient, the absence of a frame, the preservation of hair, and minimal scalp disruption may improve comfort and cosmetic satisfaction in neuro-oncological care, although patient-reported outcomes were not formally assessed.

### 3.3 Limitations and learning curve

This report reflects the initial experience of a single centre and is inevitably influenced by careful patient selection. In this context, ‘patient selection’ primarily refers to operating-room feasibility (comfortable head positioning with truly orthogonal AP/lateral fluoroscopy and uninterrupted optical tracking), rather than lesion-specific exclusion criteria.

Robotic precision depends on rigid head fixation, adequate optical tracking, and a disciplined workflow, as events such as dynamic reference base displacement or suboptimal fluoroscopic views can compromise procedure performance.

Access to dedicated robotic platforms is still limited in many institutions, which currently restricts the generalizability of this approach.

However, published series and our own experience suggest that, for teams familiar with neuronavigation, the learning curve is promising once the key steps are standardized, making the robotic workflow quickly intuitive.

### 3.4 Pearls & pitfalls

• Plan intelligent trajectories: short trajectories that are as perpendicular as possible to the skull, avoiding sulci, blood vessels and eloquent areas, without compromising surgeon comfort.
• Prioritise 2D registration quality: plan patient positioning to easily obtain high-quality orthogonal fluoroscopic views. Only accept Merge Images with excellent anatomical concordance and repeat the process if there is any doubt.
• Confirm OR geometry and optical tracking: verify C-arm sweep, robotic arm reach and uninterrupted line of sight to the DRB/end-effector/instruments; avoid mechanical conflicts.
• Control depth and calibration: calibrate drill length and EE top-to-entry distance according to skull thickness; allow just enough extra depth to traverse bone and dura without overpenetration.
• Standardised sampling: obtain multiple cores from the planned target region and remain alert for intraoperative bleeding along the biopsy tract.
**•**Robot ≠ autopilot: maintain standard stereotactic vigilance and preparedness for complications; do not let robotics replace clinical judgement.

Taken together, these elements support the ExcelsiusGPS-guided stereotactic biopsy workflow described here as a safe, accurate, pragmatic and reproducible option in appropriately selected patients, provided that a rigorous, standardized protocol is followed. Larger series will better define its role alongside established stereotactic techniques.

## Ethical approval and consent

All procedures were conducted in accordance with the Declaration of Helsinki and relevant institutional guidelines. Ethical review and approval were waived in accordance with institutional and national requirements, as this technical note describes standard clinical practice using fully anonymized clinical data and media with no patient identifiers. Written informed consent for the use of clinical information, images and videos for scientific and educational purposes was obtained from the patient.

## Author contributions (CRediT roles)

All authors have read and approved the final manuscript.

Mario Taravilla-Loma (MTL): Conceptualization; Methodology; Investigation (second surgeon); Data curation; Visualization; Writing – original draft; Writing – review & editing. MTL served as the corresponding author.

Víctor Rodríguez-Domínguez (VRD): Methodology; Investigation (principal surgeon); Validation; Supervision; Writing – review & editing.

Catalina Vivancos Sánchez (CVS): Investigation (third surgeon); Validation; Supervision; Writing – review & editing.

María Luisa Gandía-González (MLGG): Validation; Supervision; Project administration; Writing – review & editing.

Alberto Isla Guerrero (AIG): Validation; Supervision; Project administration; Writing – review & editing.

## Declaration of generative AI and AI-assisted technologies in the manuscript preparation process

During the preparation of this work the author(s) used ChatGPT/OpenAI in order to assist in language editing, structural refinement, and consistency checking of this manuscript. After using this tool/service, the author(s) reviewed and edited the content as needed and take(s) full responsibility for the content of the published article.

## Funding

This research received no specific grant from funding agencies in the public, commercial, or not-for-profit sectors.

## Declaration of competing interest

All authors declare that they have no known competing financial interests or personal relationships that could have appeared to influence the work reported in this paper.

## Data Availability

All data supporting the findings of this technical note are included within the article and its supplementary materials (figures and videos). Additional information is available from the corresponding author on reasonable request.

## References

[bib1] Bex A., Mathon B. (2023).

[bib2] Chuck C., Ali R., Lee C.K., Malik A.N., Svokos K.A., Cielo D. (2024). Neuro-oncology application of next-generation, optically tracked robotic stereotaxis with intraoperative computed tomography: a pilot experience. Neurosurg. Focus.

[bib3] Feng Y., Yaming W., Yongzhi S., Penghu W., Hong W., Xiaotong F. (2023). Novel application of robot-guided stereotactic technique on biopsy diagnosis of intracranial lesions. Front. Neurol..

[bib4] Gecici N.N., Hameed N.U.F., Habib A., Deng H., Lunsford L.D., Zinn P.O. (2025). Comparative analysis of efficacy and safety of frame-based, frameless, and robot-assisted stereotactic brain biopsies: a systematic review and meta-analysis. Oper. Neurosurg..

[bib5] Hu Y., Cai P., Zhang H., Adilijiang A., Peng J., Li Y. (2022). A comparation between frame-based and robot-assisted in Stereotactic Biopsy. Front. Neurol..

[bib6] Li C., Wu S., Huang K., Li R., Jiang W., Wang J. (2023). A comparison of the safety, efficacy, and accuracy of frame-based versus Remebot robot-assisted stereotactic systems for biopsy of brainstem tumors. Brain Sci..

[bib7] Li Y., Wu D., Yan F., Wei P., Wang W., Wang Y. (2025). Analysis of factors influencing diagnostic yield and target point error in robot-assisted stereotactic brain biopsy: a single-center experience. Neurosurg. Rev..

[bib8] Mallereau C.H., Chibbaro S., Todeschi J., Mathon B., Biopsies Brain (2025).

[bib9] Mathon B., Riche M., Lombard A., Chabaane M., Roblot P., Boetto J., Mathon B. (2025).

[bib10] Porto Junior S., Meira D.A., da Cunha B.L.B., Fontes J.H.M., Pustilnik H.N., Medrado Nunes G.S. (2024).

